# Synthetic oligonucleotides as quantitative PCR standards for quantifying microbial genes

**DOI:** 10.3389/fmicb.2023.1279041

**Published:** 2023-10-24

**Authors:** Xingguo Han, Karin Beck, Helmut Bürgmann, Beat Frey, Beat Stierli, Aline Frossard

**Affiliations:** ^1^Forest Soils and Biogeochemistry, Swiss Federal Institute for Forest, Snow and Landscape Research (WSL), Birmensdorf, Switzerland; ^2^Eawag, Swiss Federal Institute of Aquatic Science and Technology, Kastanienbaum, Switzerland

**Keywords:** synthetic DNA, plasmid, real-time quantitative PCR, qPCR standards, qPCR methodology, microbial and functional gene abundances, C and N cycling

## Abstract

Real-time quantitative PCR (qPCR) has been widely used to quantify gene copy numbers in microbial ecology. Despite its simplicity and straightforwardness, establishing qPCR assays is often impeded by the tedious process of producing qPCR standards by cloning the target DNA into plasmids. Here, we designed double-stranded synthetic DNA fragments from consensus sequences as qPCR standards by aligning microbial gene sequences (10–20 sequences per gene). Efficiency of standards from synthetic DNA was compared with plasmid standards by qPCR assays for different phylogenetic marker and functional genes involved in carbon (C) and nitrogen (N) cycling, tested with DNA extracted from a broad range of soils. Results showed that qPCR standard curves using synthetic DNA performed equally well to those from plasmids for all the genes tested. Furthermore, gene copy numbers from DNA extracted from soils obtained by using synthetic standards or plasmid standards were comparable. Our approach therefore demonstrates that a synthetic DNA fragment as qPCR standard provides comparable sensitivity and reliability to a traditional plasmid standard, while being more time- and cost-efficient.

## 1. Introduction

Soil microorganisms are a critical component of the Earth system by contributing significantly to global elemental cycles through a complex network of biogeochemical reactions (Schimel and Schaeffer, 2012). In many ecosystems, microorganisms gain energy for growth and survival through degrading organic matter (OM) to carbon dioxide (CO_2_, under both aerobic and anaerobic conditions), methane (CH_4_, under anaerobic conditions), and nitrous oxide (N_2_O, under aerobic and low-oxygenic conditions), releasing these greenhouse gasses to the atmosphere (Canfield et al., 2005; Oertel et al., 2016; Hutchins and Capone, 2022). Therefore, quantifying microbial abundance (as a proxy for biomass) is crucial to assess the importance of the microorganisms and understand their role or functions in ecosystems.

Over the past few decades, numerous techniques have been employed to quantify the population size of specific microorganisms or groups of microorganisms in environmental samples or synthetic communities in microbial ecology. These include, but are not limited to, direct epifluorescence microscopy (EFM) (Caron, 1983; Kepner and Pratt, 1994), flow cytometry (FCM) (Frossard et al., 2012, 2016; Deng et al., 2019), fluorescence *in situ* hybridization (FISH) (Bouvier and del Giorgio, 2003), catalyzed reporter deposition-FISH [CARD-FISH, Schippers et al. (2005), Eickhorst and Tippkotter (2008)], phospholipid quantification (Phospholipid-derived fatty acids, PLFAs) (White et al., 1979; Frostegard et al., 1991), droplet digital polymerase chain reaction (ddPCR) (Kim et al., 2014), and real-time quantitative PCR (qPCR) (Smith and Osborn, 2009; Brankatschk et al., 2012; Hartmann et al., 2014; Han et al., 2016, 2020). Additionally, there are other techniques, e.g., quantitative reverse transcription PCR (qRT-PCR) (Freeman et al., 1999), quantitative stable isotope probing (qSIP) (Hungate et al., 2015), and quantitative bioorthogonal noncanonical amino acid tagging (QBONCAT) (Bagert et al., 2014), highly used for gene expression, taxon-specific population and protein detection, respectively.

Among these approaches, qPCR has been widely used in molecular biology, as this method has proved to be relatively cheap, straightforward, and efficient with a high sensitivity, covering a linear range over 7–8 orders of magnitude, and high throughput, although it has limitations by not targeting active community members (Fierer et al., 2005). qPCR relies on optical reporter systems, either using a double-stranded DNA-binding fluorescent dye such as SYBR® Green or DNA probes dual-labeled with reporter dyes and quenchers, such as TaqMan™ probes (Orlando et al., 1998; Arya et al., 2005; Van Guilder et al., 2008). Alongside measuring the abundance of the bacterial, archaeal and fungal communities (using general bacterial, archaeal or universal primers for the 16S rRNA gene (Takai and Horikoshi, 2000) or of the ITS region for fungi (Fierer et al., 2005), qPCR has been applied for detecting and quantifying copy numbers of microbial functional genes involved in C and N cycling. Among the functions frequently studied in diverse environments using qPCR are CH_4_ production (methyl coenzyme M reductase A: *mcr*A) and oxidation (particulate methane monooxygenase: *pmo*A), nitrogen fixation (nitrogenase: *nif*H), ammonia oxidation (archaeal and bacterial ammonia monooxygenase: *amo*A), nitrite reduction (nitrite reductase: *nir*S and *nir*K), nitrite oxidation (beta subunit of nitrite oxidoreductase: *nxr*B), N_2_O production (nitric oxide reductase: *nor*B) and reduction (nitrous oxide reductase: *nos*Z), and organic phosphorus (P) hydrolysis (alkaline phosphatase D: *pho*D) (Church et al., 2005; Henry et al., 2006; Leininger et al., 2006; Steinberg and Regan, 2008; Han et al., 2016; Webster et al., 2016; Luo et al., 2017; Han et al., 2020; Perez-Mon et al., 2022).

In spite of the advantage of being a straightforward method not including too many steps, qPCR has a major drawback. To quantify a specific gene, qPCR assays require the corresponding standard for calibration under the Minimum Information for Publication of Quantitative Real-Time PCR Experiments (MIQE) guidelines (Bustin et al., 2009). Classically, standards have been produced by cloning a target sequence into a plasmid, amplifying genes via PCR, using genomic DNA directly, or acquiring commercially approved biological standards (Dhanasekaran et al., 2010; Goodwin et al., 2018). However, these approaches often incur significant costs, in terms of time and money, and potentially generate contaminations, particularly when preparing multiple plasmid standards targeting different microbial genes in parallel. For instance, both PCR amplicons and plasmids need to be purified before being used, procedure which is often causing contaminations (Cimino et al., 1991). Moreover, the quantification of plasmid copies per cell was shown to be unreliable (May et al., 2015; Conte et al., 2018). In recent years, there has been a growing interest to use artificially synthesized DNA and RNA sequences as qPCR standards. Synthesizing such sequences to produce standards is considerably faster, cleaner (low contamination risk) and also less expensive (following considerable reduction of the cost of custom DNA synthesis over the years, see Carlson, 2009) compared to traditional plasmid standards (Conte et al., 2018; Xu et al., 2019). The synthetic gene fragments can be purchased in a length of 125 to 3,000 base pair (bp) with known degenerate nucleotides of A, T, C, and G (May et al., 2015; Conte et al., 2018). Up to now, most of the artificially synthesized standards have been used for medical purpose, focusing on viral or infectious microorganisms (Tourinho et al., 2015; Fesolovich and Tobe, 2017; Lima et al., 2017; Magee et al., 2017; Bandeira et al., 2020; Bivins et al., 2021; Munoz-Calderon et al., 2021), very few in environmental samples. To the best of our knowledge, the few studies using synthesized gene fragments as qPCR standards in environmental microbiology studies assessed bacterial 16S rRNA and *htrA* genes in soils (Gunawardana et al., 2014; Sirois and Buckley, 2019), 16S rRNA genes and methanogenic *mcr*A in a biogas digester (May et al., 2015), antibiotic resistance genes in environmental water, soil and faeces samples (Xu et al., 2019), and 16S rRNA genes by adding synthetic DNA internal standard to fecal samples (Zemb et al., 2020). We propose that, given the advantages, synthetic qPCR standards can and should be widely adopted for qPCR analysis of functional genes in environmental microbiology and microbial ecology. However, this new methodological approach should be thoroughly evaluated and compared to previous practice before being adopted.

Here, we designed qPCR standards for a number of frequently studied functional genes of the C, N and P cycle, and the ITS region and the 16S rRNA gene by synthesizing double-stranded DNA fragments obtained by generation of consensus sequences from alignments of microbial gene sequences. To provide a thorough evaluation of the effectiveness and reliability of synthetic DNA fragments as qPCR standards, we compared these newly synthesized qPCR standards with standards produced via plasmids in different qPCR assays, targeting several different taxonomic, and functional genes of soil microorganisms.

## 2. Materials and methods

### 2.1. Production of qPCR standards

Synthetic standards were designed for qPCR assays targeting bacterial 16S rRNA genes, fungal ITS and a broad range of genes involved in C, N and P cycling, *mcr*A, *pmo*A, *nif*H, *nos*Z, *amo*A, *nir*S, *nir*K, *nxr*B, *nor*B, and *pho*D genes (Table 1). Synthetic DNA fragments were designed by aligning between 10 and 20 gene sequences per targeted gene with the software Geneious (version 9.1.8). All gene sequences were downloaded from the National Center for Biotechnology Information (NCBI) gene database. For each targeted gene, a consensus sequence was obtained from at least 10 downloaded sequences by the “Multi Align” function in Geneious (Alignment type: Global alignment with free end gaps, Cost Matrix: 65% similarity, Gap open penalty: 12, Gap extension penalty: 3, Refinement iterations: 2). The consensus sequences were created with most frequent nucleotide for each base of the aligned sequences, containing only nucleotides of A, T, C, and G. Forward and reverse primer sequences of each targeted gene were then searched against the corresponding consensus sequence by the “Test with Saved Primers” function in Geneious (Maximum mismatches: 2), to ensure the match between primers and standard sequences of the gene of interest. For each synthetic fragment, 9 to 30 additional bases next to forward and reverse primers (flanking ends) were kept. The consensus sequences of the synthetic DNA fragments of each gene were searched and blasted against the NCBI database (Supplementary Table S1). Double-stranded synthetic DNA fragments (between 250 and 650 bp) were then ordered from gBlocks gene fragments (Integrated DNA Technologies: IDT, Inc.), with 500 ng dry DNA in a tube for each target. Upon reception, the DNA was resuspended in nuclease-free water (H_2_O) and stored at −20°C freezer for long-term use. The copy numbers of synthetic DNA per microliter were calculated using the formula according to Godornes et al., 2007:

$$\frac{\mathrm{gene}\;\mathrm{copies}}{\mathrm{\mu L}}=\frac{DNA\;\mathrm{concentration}\;\left(\mathrm{ng}/\mathrm{\mu L}\right)\times 10^{-9}\times 6.022\times 10^{23}}{\left(\mathrm{fragment}\;\mathrm{size}\;\mathrm{bp}\right)\times 660\;g/\mathrm{mol}\;}$$

where the Avogadro number is 6.022 × 10^23^ (molecules/mole), the fragment size is the length of the synthesized DNA (bp), and 660 is the average weight of a single DNA base pair (g/mol).

**Table 1:** Primers for the quantification of total abundance of bacterial 16S rRNA genes and fungal ITS2 region, *mcr*A (methyl coenzyme M reductase A: methanogenic Archaea), *pmo*A (particulate methane monooxygenase: methanotrophic Bacteria), *nif*H (nitrogenase: N fixers), *nos*Z (nitrous oxide reductase: N_2_O reducers), archaeal *amo*A and bacterial *amo*A (archaeal and bacterial ammonia monooxygenase: ammonia oxidizers), *nir*S and *nir*K (nitrite reductase: nitrite reducers), *nxr*B (beta subunit of nitrite oxidoreductase: nitrite oxidizers), *nor*B (nitric oxide reductase: N_2_O producers) and *pho*D (alkaline phosphatase D: organic phosphorus hydrolyzers) by qPCR.

Gene	Targeted phylogenetic group/Processes involved	Primers	Primer Sequence (5′-3′)	Amplicon size vs. synthetic standard length (base pair, bp)	Primers Reference
Bacterial 16S rRNA gene	Bacteria universal	349F	AGG CAG CAG TDR GGA AT	~460 vs. 498	Takai and Horikoshi (2000)
		806R	GGA CTA CYV GGG TAT CTA AT		
ITS	Fungal universal	ITS3	CAH CGA TGA AGA ACG YRG	~430 vs. 472	Frey et al. (2016), Tedersoo et al. (2014)
		ITS4	TCC TSC GCT TAT TGA TAT GC		
*mcr*A	Methane production	ML-F(mcrA)32	GGT GGT GTM GGA TTC ACA CAR TAY GCW ACA GC	~476 vs. 500	Luton et al. (2002)
		ML-R(mcrA)23	TTC ATT GCR TAG TTW GGR TAG TT		
*pmo*A	Methane oxidation	A189F	GGN GAC TGG GAC TTC TGG	~532 vs. 572	Holmes et al. (1995)
		A682r	GAA SGC NGA GAA GAA SGC		
*nif*H	Nitrogen fixation	PolF_115	TGC GAY CCS AAR GCB GAC TC	~362 vs. 416	Poly et al. (2001)
		PolR_457	ATS GCC ATC ATY TCR CCG GA		
*nos*Z	N_2_O reduction	nosZ-1F	WCS YTG TTC MTC GAC AGC CAG	~249 vs. 309	Henry et al. (2006)
		nosZ_1R	ATG TCG ATC ARC TGV KCR TTY TC		
archaeal *amo*A	Ammonia oxidation	Arch-amoAF	CTG AYT GGG CYT GGA CAT C	~635 vs. 665	Francis et al. (2005)
		Arch-amoAR	TTC TTC TTT GTT GCC CAG TA		
bacterial *amo*A	Ammonia oxidation	amoA-1F	GGG GHT TYT ACT GGT GGT	~491 vs. 531	Rotthauwe et al. (1997)
		amoA-2R	CCC CTC KGS AAA GCC TTC TTC		
*nirS*	Nitrite reduction	cd3AF	GTS AAC GTS AAG GAR ACS GG	~425 vs. 465	Throback et al. (2004)
		R3cd	GAS TTC GGR TGS GTC TTG A		
*nirK*	Nitrite reduction	nirK_F1aCu	ATC ATG GTS CTG CCG CG	~473 vs. 513	Hallin and Lindgren (1999)
		nirK_R3Cu	GCC TCG ATC AGR TTG TGG TT		
*nxrB*	Nitrite oxidation	nxrB169f	TAC ATG TGG TGG AAC A	~485 vs. 525	Pester et al. (2014)
		nxrB638r	CGG TTC TGG TCR ATC A		
*nor*B	N_2_O production	qnorB2F	GGN CAY CAR GGN TAY GA	~260 vs. 284	Braker and Tiedje (2003)
		qnorB5R	ACC CAN AGR TGN ACN ACC CAC CA		
*pho*D	Organic phosphorus hydrolysis	phoD-F733	TGG GAY GAT CAY GAR GT	~363 vs. 377	Ragot et al. (2015)
		phoD-R1083	CTG SGC SAK SAC RTT CCA		

The top six genes were compared with both synthetic and plasmid standards.

For several targeted genes, qPCR standards were also produced via plasmids. PCR products of the particular gene (bacterial 16S rRNA gene, fungal ITS region, *mcr*A, *pmo*A, *nif*H, and *nos*Z) were cloned into the vector and competent cells using the pGEM-T Easy Vector System II Systems Kit according to the manufacturer’s instructions (Promega, Madison, WI, United States) (Henry et al., 2006; Frey et al., 2011). Briefly, PCR reactions were conducted to amplify the targeted gene from DNA extracted from soils. PCR products were then inserted to the Vector (Ligation), which was added to the *E. coli* JM109 competent cells (Transformation). Transformed *E. coli* were then spread on Luria-Bertani (LB) agar plates with appropriate antibiotics. After 16–24 h of incubation at 37°C, colonies were observed on the plates, and only white colonies were picked up and incubated in liquid LB medium to grow with shaking over 24 h. The plasmids were extracted by Plasmid Miniprep Kit (Promega, Madison, WI, United States) according to the manufacturer’s instructions. The plasmids were further verified by Sanger sequencing: Colony PCR products of the selected marker genes were sequenced on both strands (up to 960 bp), according to Frey et al. (2008). Cycle sequencing was carried out using the Big Dye-Terminator Cycle Sequencing Kit, version 1.3 (PE Applied Biosystems, Foster City, CA) according to the manufacturer’s recommendations. The copy numbers of plasmid DNA per microliter were calculated using the following formula (Godornes et al. (2007):

$$\frac{\mathrm{gene}\;\mathrm{copies}}{\mathrm{\mu L}}=\frac{DNA\;\mathrm{concentration}\;\left(\mathrm{ng}/\mathrm{\mu L}\right)\times 10^{-9}\times 6.022\times 10^{23}}{\left(3015\;\mathrm{bp}+\mathrm{amplicon}\;\mathrm{size}\;\mathrm{bp}\right)\times 660\;g/\mathrm{mol}\;}$$

where the length of the pGEM-T Easy vector is 3,015 bp.

Standards produced by synthetic DNA and plasmids were directly compared by qPCR (top six genes in Table 1). Synthetic DNA fragments for microbial functional genes *amo*A, *nir*S, *nir*K, *nxr*B, *nor*B and *pho*D, were not compared with homologous standards produced via cloning, but were tested and verified by qPCR assays.

### 2.2. qPCR of standards and soil DNA

The effectiveness of the standard fragments produced via synthetic DNA and plasmids clones were tested and compared in qPCR assays for different genes with soil DNA on a QuantStudio5 Real-Time PCR System (Thermo Fisher Scientific, Waltham, MA, United States) by SYBR Green, which has been widely applied in soils and freshwater ecosystems (Stubner, 2002; Han et al., 2016, 2020). qPCR reactions (10 μL) were composed of 5 μL GoTaq^®^ qPCR Master Mix (Promega, Madison, WI, United States), 0.1 μL 30 mg mL^−1^ bovine serum albumin (BSA), 0.5 μL 10 μM of each primer, 1.9 μL molecular-grade water and 2 μL DNA template. Soil DNA was diluted at a concentration of ~2 ng/μL to avoid potential PCR inhibition. The different primers used in the reactions are shown in Table 1 and details on qPCR thermocycling conditions are described in Table 2. Three standard dilution series per target gene (for both synthetic DNA and plasmid DNA standards) were obtained from 10-fold serial dilutions of standards with molecular-grade H_2_O. The standard series ranged ranging from 10^1^ to 10^8^ copies per μL.

**Table 2:** qPCR thermocycling conditions for the quantification of each target gene.

	**16S rRNA gene**	**ITS**	***mcr*A**	***pmo*A**	***nif*H**	***nos*Z**	archaeal *amo*A	bacterial *amo*A	*nir*S	*nir*K	*nxr*B	*nor*B	*pho*D
Stage 1: 1 cycle (denaturation)	2 min (95°C)												
Stage 2: 40 cycles (amplification)	40s (95°C) 40s (53°C) 1 min (72°C)	40s (95°C) 40s (58°C) 1 min (72°C)	45 s (95°C) 45 s (50°C) 45 s **(72°C)**	45 s (95°C) 45 s (56°C) 45 s **(72°C)**	45 s (95°C) 45 s (55°C) 30s **(72°C)**	45 s (95°C) 45 s (55°C) 45 s **(72°C)**	45 s (95°C) 45 s (53°C) 45 s (72°C)	45 s (95°C) 45 s (55°C) 45 s (72°C)	45 s (95°C) 45 s (58°C) 45 s (72°C)	45 s (95°C) 45 s (57°C) 45 s (72°C)	45 s (95°C) 45 s (57°C) 45 s (72°C)	45 s (95°C) 45 s (57°C) 45 s (72°C)	30s (95°C) 30s (58°C) 30s (72°C)
Stage 3: 1 cycle (melting curve)	15 s (95°C) 15 s (60°C) 15 s (95°C)	15 s (95°C) 15 s (60°C) 15 s (95°C)	15 s (95°C) 15 s (50°C) 15 s (95°C)	15 s (95°C) 15 s (60°C) 15 s (95°C)	15 s (95°C) 1 min (50°C) 15 s (95°C)	15 s (95°C) 1 min (50°C) 15 s (95°C)	15 s (95°C) 15 s (60°C) 15 s (95°C)	15 s (95°C) 15 s (60°C) 15 s (95°C)	15 s (95°C) 15 s (60°C) 15 s (95°C)	15 s (95°C) 15 s (60°C) 15 s (95°C)	15 s (95°C) 15 s (60°C) 15 s (95°C)	15 s (95°C) 15 s (55°C) 15 s (95°C)	15 s (95°C) 15 s (60°C) 15 s (95°C)
Stage 4: cooling	4°C												

The first six genes were compared with both synthetic and plasmid standards.

The soils used in this study were collected in Switzerland in August 2021. The five soil samples were collected from the top 10 cm along an altitudinal gradient (Supplementary Table S2). The soils varied in DNA concentration, pH, total carbon (TC), organic C (TOC) and nitrogen (TN) content (Supplementary Table S2). Soil pH was measured in 0.01 M CaCl_2_ in soil-solution ratio of 1:2 (dry weight/volume) with a pH meter. TC and TN were measured on dried (60°C) and fine-grained soils by an elemental analyzer (NC-2500; CE Instruments, Wigan, United Kingdom). Soil TOC was quantified after HCl-fumigation using an elemental analyzer (Walthert et al., 2010). Total DNA was extracted from 0.25–0.30 g soil with the DNeasy Powersoil Pro Kit (Qiagen, Hilden, Germany) according to the manufacturer’s instructions. The DNA was quantified with PicoGreen (ThermoFisher Scientific, Cleveland, OH, United States), and subsequently diluted to 2.0 ng per μL by molecular-grade H_2_O and measured in triplicate.

A standard curve for each gene was generated by plotting cycle threshold (Ct) or quantification (Cq) of cycle values of each dilution step against the corresponding log10 transformed number of gene copies in the standard. The amplification efficiency (*E*) was estimated using the slope of the standard curve with the formula: *E* = (10^−1^/slope) − 1. The detection limit was 10 copies per μL according to the lowest concentration standard (10^1^ copies per μL).

## 3. Results and discussion

### 3.1. Performance of synthetic and plasmid DNA standards

**Figure 1:**
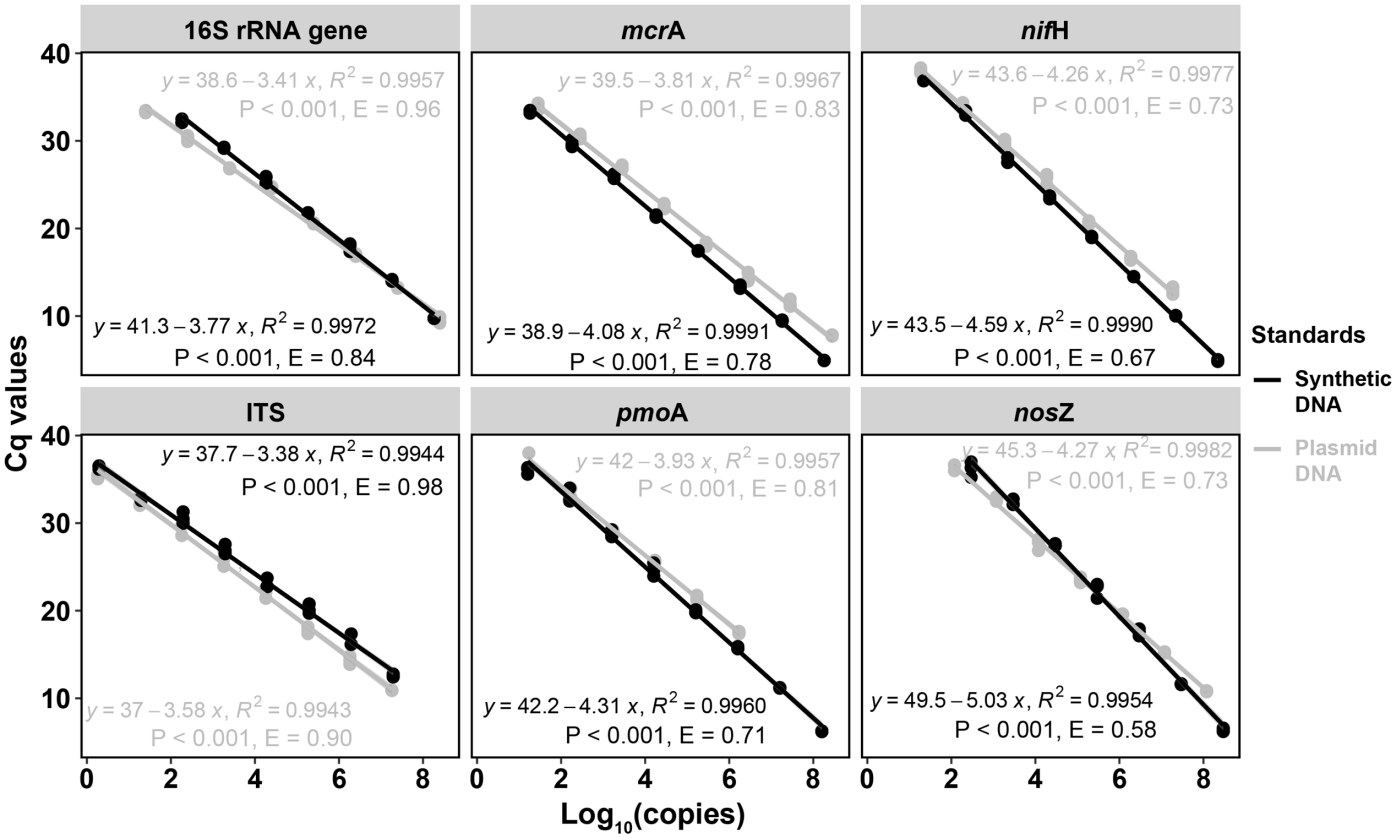
Comparisons of qPCR standard curves between synthetic DNA and plasmid DNA standards. Standards were diluted by 10 times for each step from 10^8^ to 10^1^ copies per μL. *R*^2^ is the coefficient of determination. *p* < 0.001 indicates the significance of the linear regression. Gene copy numbers (copies per dry gram soil) were log10-transformed. *E* = (10–1/slope) – 1.

**Figure 2:**
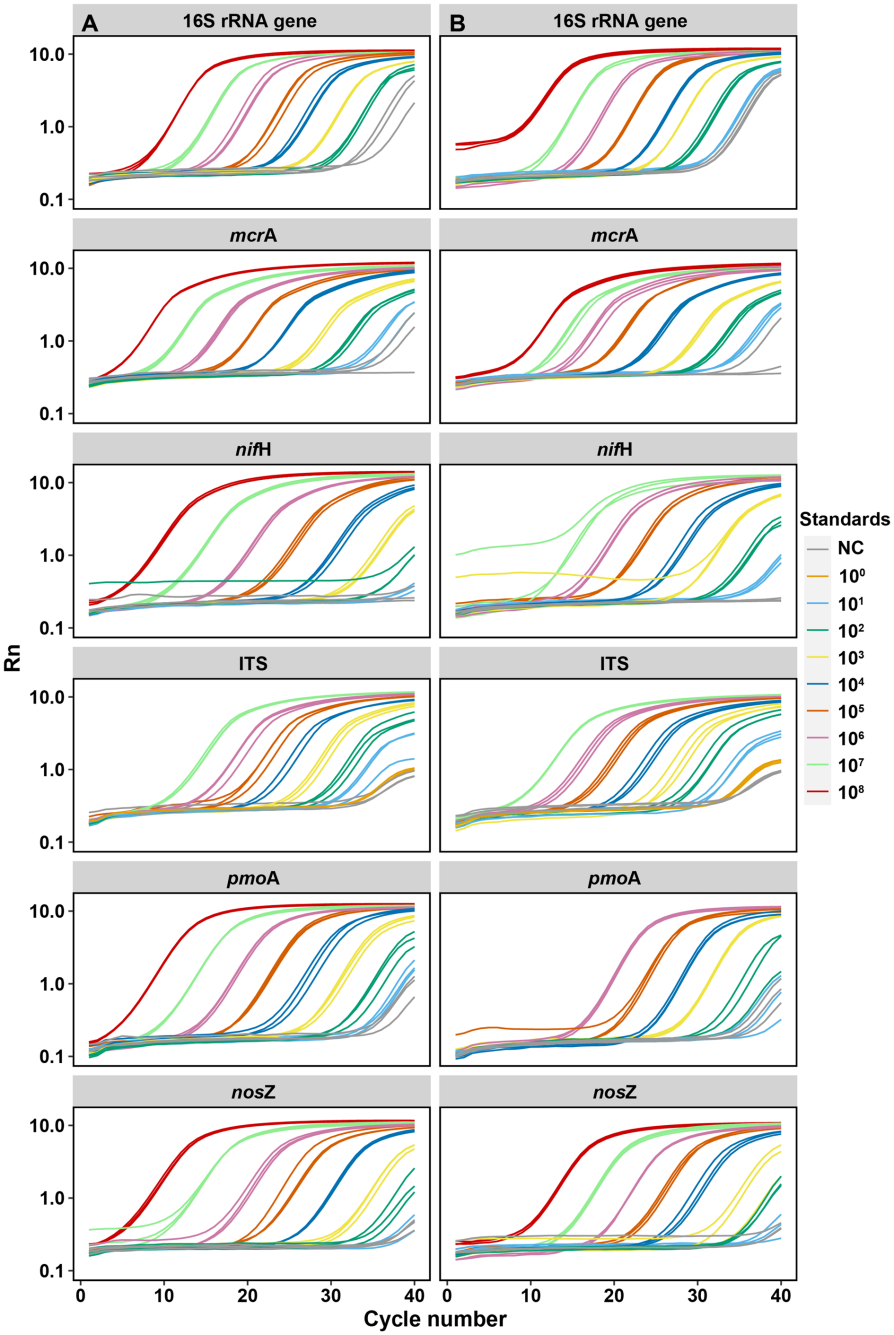
Amplification curves of each target gene by serial dilutions between synthetic DNA [column **(A)**] and plasmid DNA [column **(B)**] standards. Each qPCR reaction (10 μL total) contained 2 μL of DNA template (standard). Standards were diluted by 10 times for each step from 10^8^ to 10^1^ copies per μL. NC: negative control. Rn is the fluorescence of the reporter dye divided by the fluorescence of a passive reference dye; i.e., Rn is the reporter signal normalized to the fluorescence signal of Applied Biosystems™ ROX™ Dye.

For the six targeted genes which were tested with synthetic DNA and plasmid DNA standards (Table 1), both standards for qPCR quantifications yielded significantly (*p* < 0.001) linear calibration curves featuring a coefficient value (*R*^2^) of >0.99 (Figure 1), together with similar high R^2^ derived from the genes tested with synthetic DNA standards (Supplementary Figure S1). The dilution series of both synthetic DNA and plasmid DNA standards exhibited smooth and exponential amplification curves (Figure 2). Coefficients of variation of Cq values among the replicates for the standards in the range from 10^1^ to 10^8^ copies per μL were between 0.03 and 4.15%, which indicated good repeatability and reproducibility. Additionally, the slopes of the synthetic standards were similar to those of the plasmid standards, with only minor differences (Figure 1). Altogether, provided compelling evidence that, similar to traditional plasmid standards, synthetic DNA standards of serial dilutions can be amplified effectively and produce high-quality and consistent standard curves. Comparable standard curves between synthetic DNA standards and traditional standards (PCR amplicons and plasmids of cloning) have been reported in previous studies targeting human mitochondrial gene (Conte et al., 2018), antibiotic resistance genes (*erm*B for macrolides) from water, soil and faeces (Xu et al., 2019), human T-cell leukemia virus type 1 (HTLV-1) (Bandeira et al., 2020) and Hepatitis B virus (HBV) (Portilho et al., 2018). Yet, to the best of our knowledge, this is the first study to use synthetically designed and produced DNA fragments as qPCR standards targeting a wide range of genes involved in C and N cycling employed in microbial ecology.

Standard curves from both synthetic and plasmid standards showed high and similar amplification efficiency (E) values, confirming the reliability of synthetic gene fragments as qPCR standards (Figures 1, 2). PCR efficiency of the standard curves for 16S rRNA gene reached 0.84 for synthetic DNA and 0.96 for plasmid standards. It attained 0.98 and 0.90 for fungal ITS region for the synthetic and plasmid standard, respectively. Efficiency values of the remaining four genes tested (*mcr*A, *pmo*A, *nif*H, and *nos*Z), were all lower than 0.90, irrespective of plasmid or synthetic standards (Figure 1). Ideally, an efficiency value over 0.90 is considered a well amplified standard and a qualified standard curve (Svec et al., 2015). However, due to the potential PCR self-inhibition, caused by reagent limitation, accumulation of pyrophosphate molecules, self-annealing of accumulating products, polymerase and protein inhibition, or primer specificity and contamination, E values can be as low as 0.70 (Luby et al., 2016; Xu et al., 2019). The slightly lower E value for synthetic 16S rRNA gene standard during PCR might be caused by a small peak (PCR byproduct) right before the main PCR product peak of 16S rRNA gene, especially the least diluted ones, implied by the melting curves (Supplementary Figure S2A), which caused the differences in the standard curves from synthetic and plasmid standards. Lower E values of the genes *mcr*A, *pmo*A, *nif*H and *nos*Z from both synthetic and plasmid standards were likely also related to PCR inhibitions, in particular to the least diluted standards. Usually, 3.3 (a slope of – 3.3) cycles apart of the 10-fold dilutions were considered as an indicator of 100% PCR efficiency (Svec et al., 2015). However, much higher Cq value differences between the dilution series were found for these four genes from both standards (Figure 1), indicating an inhibition effect. Additionally, different instruments and volume for standard dilution also can affect PCR efficiency. A larger pipeted volume (for example 10 μl) transfered across dilutions could increase the efficiency of the qPCR (Svec et al., 2015), however only 2 μl was used in this study.

In spite of the similarity of qPCR standard curves between synthetic and plasmid standards, there were slight differences in the slopes and E values of standard curves between these two standards (Figure 1). For *mcr*A, *pmo*A, *nif*H, and *nos*Z, standard curves from synthetic standards were always steeper (higher absolute slopes) than those from plasmid standards, with Cq values of the least diluted standards from synthetic standards lower than those of the least diluted plasmid standards, even when the copy numbers of the least diluted standards from synthetic standards were lower than those from plasmid standards for *mcr*A and *pmo*A (Supplementary Table S3). This indicated an inhibition of the least diluted plasmid standards, which took more cycles (higher Cq values) to get fully amplified. For ITS region, standard curve from plasmid standard was slightly steeper than that from synthetic standard, which also indicated an effect of inhibition. In addition to PCR self-inhibition, there might be also a conformation effect of non-linear plasmid standards, which could overestimate qPCR results. Large quantification bias by plasmid DNA conformation was found with significant differences between circular and linear plasmid standards (Lin et al. (2011). Moreover, measuring the abundance of microalgal proliferating cell nuclear gene (*pcna*) by qPCR, Hou et al. (2010) found a high overestimation of *pcna* gene copies when using circular plasmid standard in comparison to a linear one. However, similar gene estimates were observed from circular and linear plasmid standards for quantifying prokaryotic 16S rRNA gene by qPCR assays (Oldham and Duncan, 2012). Therefore, the slight differences between synthetic and plasmid standards curves in this study might be partially caused by standard DNA conformation.

### 3.2. Microbial gene abundances in soils based on synthetic and plasmid DNA standards

**Figure 3:**
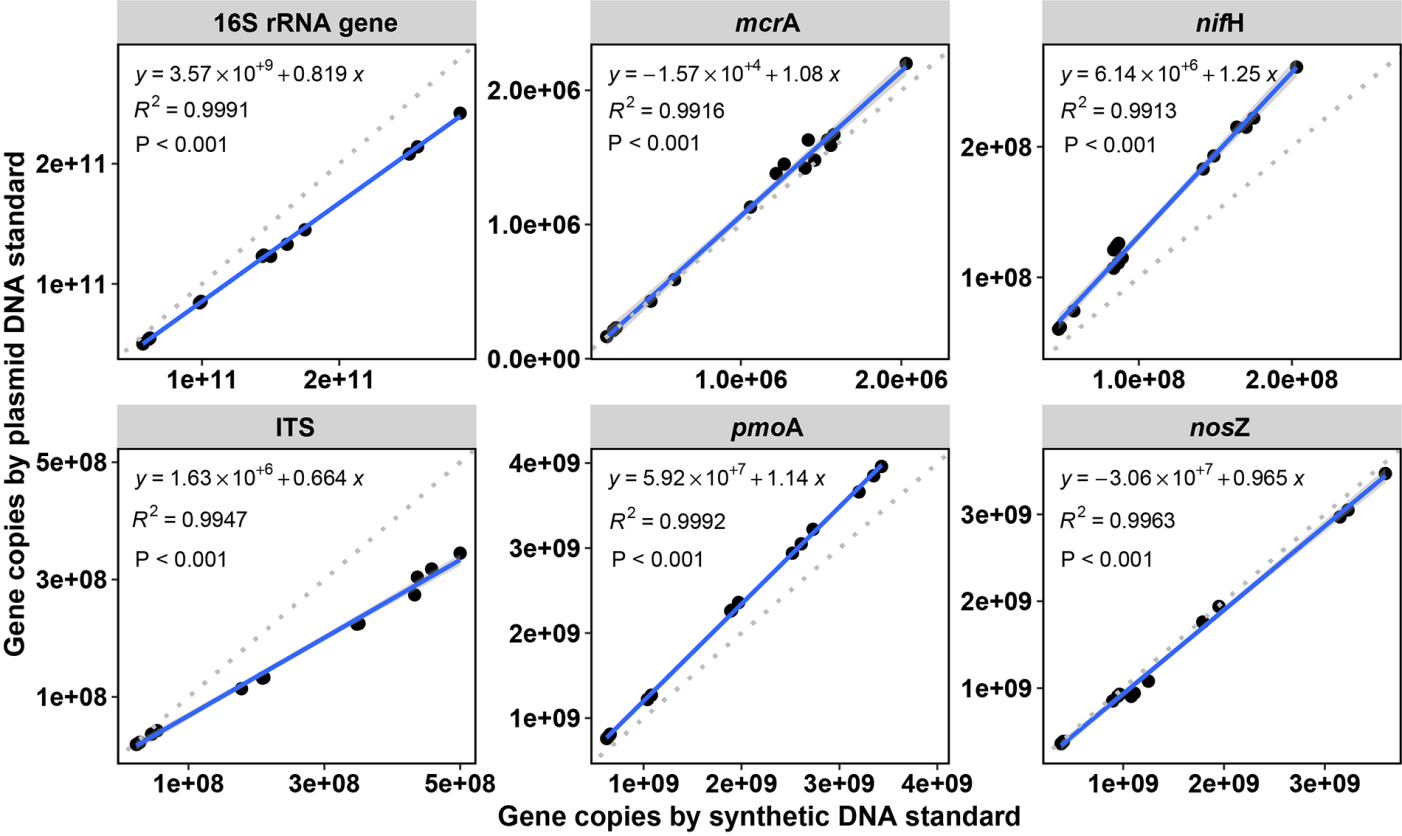
Linear correlations between copy numbers in soil samples (copies per dry gram soil) of the tested genes by qPCR between synthetic DNA and plasmid DNA standards. *R*^2^ is the coefficient of determination. *p* < 0.001 indicates the significance of the linear regression. The gray dashed lines are 1:1 reference lines.

**Figure 4:**
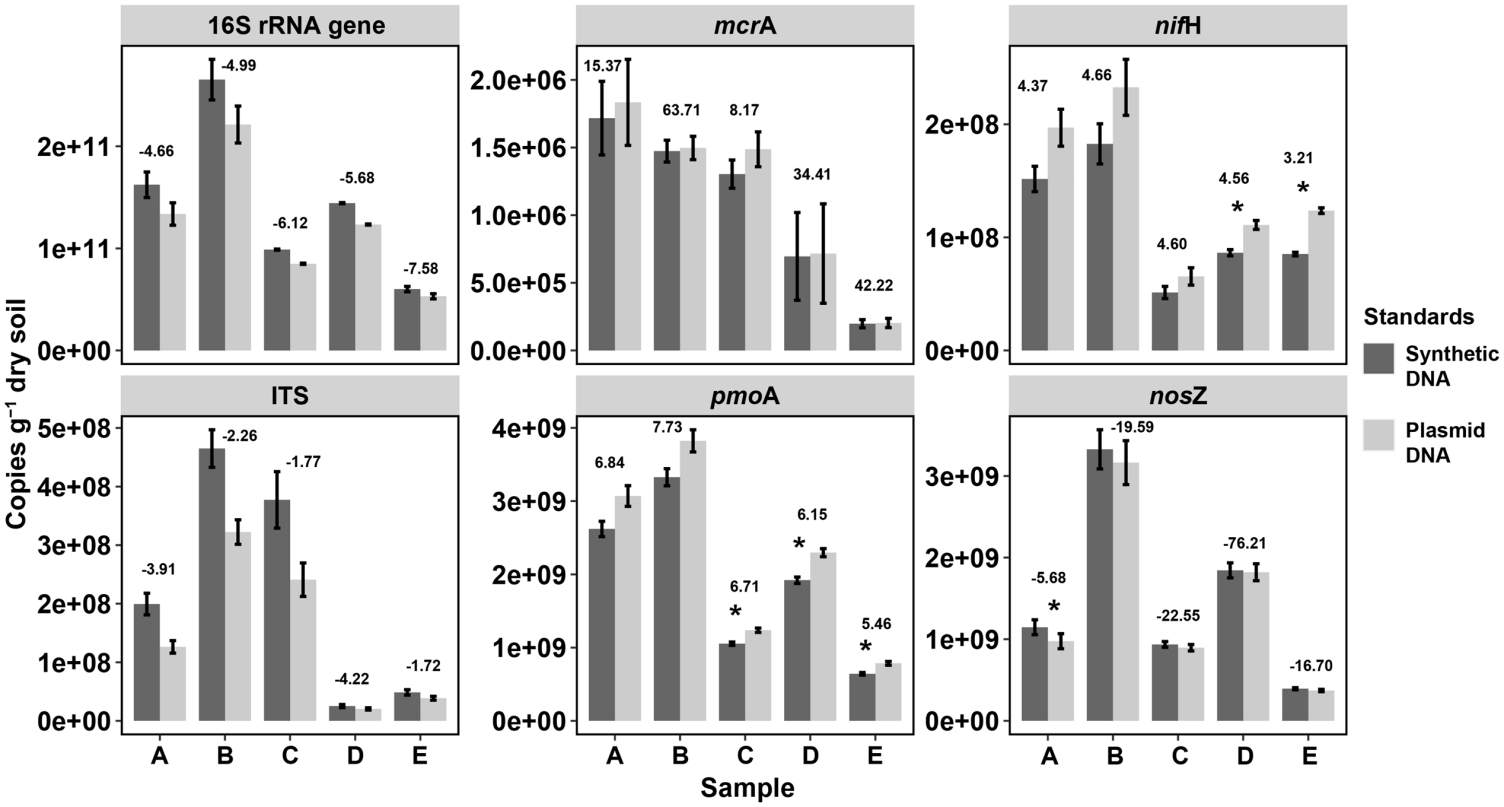
Comparisons of gene copy numbers in soils by qPCR between synthetic and plasmid DNA standards. Significant differences in gene copies between plasmid DNA and synthetic DNA standards are marked as asterisks (*p < 0.05) based on *T*-test by the function compare_means() from the R package ggpubr. *p*-values were adjusted by the “Bonferroni” method. Values above the two bars at each elevational site indicate the delta value of the average: mean (copies by plasmid standard)/[mean (copies by plasmid standard) – mean (copies by synthetic standard)/]. The larger the absolute delta values, the more similar copy numbers of the tested genes per soil sample from the two qPCR standards.

In order to validate the reliability of our synthetic DNA standards, the abundances of the tested genes were quantified in DNA extracts from soils by qPCR assays and the gene copy abundance calculated using standards from synthetic and plasmid DNA (Figure 3). Little amplification was observed in negative control (NC) reactions. The amplification in the no template controls is likely an unintended byproduct driven by the overly manipulative chemistry being exerted during 40 cycles (McCall et al., 2014), probably also due to contamination of reaction preparation (preparation of replicates, dilutions, pipetting, etc.) (Svec et al., 2015). Overall, gene copies calculated with either standard curves (i.e., from synthetic or plasmid DNA) were not significantly different for all the genes studied across all soil samples except for few assays (6 of 30 samples across 6 tested genes, marked with asterisks in Figure 4). These significant differences reflect the described differences of the standards curves thus that gene copy numbers calculated from synthetic standards were on average lower than those from plasmid standards (difference of 5.1 ± 4.4% for *mcr*A, 15.4 ± 1.2% for *pmo*A, 23.8 ± 4.1% for *nif*H, and 6.9 ± 6.2% for *nos*Z) or higher for 16S rRNA gene (17.7 ± 3.2%) and ITS region (41.6 ± 16.4% higher). Gene copies varying within one log (10 times) were widely reported for qPCR quantifications of viruses with synthetic and plasmid standards, and such results have been considered good agreement of the two methods (Tourinho et al., 2015; Lima et al., 2017; Portilho et al., 2018; Bandeira et al., 2020).

Despite the described deviation, correlation of gene copies in the soil samples using the synthetic standard and copies by using plasmid standard, was significant with a squared coefficient (R^2^) of over 0.99 (Linear correlations, all significant *p* < 0.001) for all the six tested genes (Figure 3), which showed highly identical results with both standards. Similarly, high R^2^ (0.83) based on linear correlations were also observed in a study on human virus by qPCR when comparing synthetic DNA and plasmid DNA standards (Bandeira et al., 2020). Furthermore, when comparing the variation in gene copy numbers among soils normalized to the soil with the highest copy numbers (for each gene, Supplementary Figure S3), no significant difference was observed between synthetic and plasmid DNA standards. This further showed the high identity of the two standards of qPCR in quantifying soil DNA. Therefore, considering the sensitivity and efficiency of qPCR, inhibition and human errors (i.e., pipetting errors), differences in copy numbers within 50% variation in this study are very much acceptable, especially with gene concentrations reaching up to more than 10^10^ copies per dry gram soil.

Taken all together, our results demonstrated that the synthetic DNA standards are reliable for qPCR quantification of various phylogenetic and functional genes in soils. Furthermore, we could validate the usage of synthetic DNA standards designed from consensus sequences of 10 to 20 aligned microbial gene sequences. We however recognized that 10 to 20 sequences may be a limited number for consensus sequence design given what is known for the specific genes studied here and their diversity. Moreover, we suggest to include at least 10 additional bases at each flanking end of the targeted gene primer consensus sequence, to assure that the target gene can be completely amplified. As a future development perspective, multiplexing multiple targeted genes on a single gBlocks fragment (i.e., designing one standard sequence that can be used in qPCR assays of several targeted genes) could be developed, under the condition that randomly nucleotides are inserted as placeholders (spacer) between the primers binding position to assure matching distances (May et al., 2015). Finally, creating a repository where all designed consensus sequences targeting a variety of phylogenetic and functional genes could be deposited, would greatly ease the dissemination and accessibility of information needed to synthesize and broaden the use of synthesized standards for qPCR assays.

## 4. Conclusion

In this study, we designed qPCR standards for quantifying various phylogenetic and functional genes used in microbial ecology, such as those involved in C and N cycling, by synthesizing double-stranded DNA sequences as gBlocks gene fragments. We show that synthetic DNA standards performed equally well as traditional plasmid standards in producing linear qPCR calibration curves, yielding precise and efficient results for a broad range of soils. The application of synthetic DNA standards for qPCR assays is however not limited to soils, but can be recommended for all kind of genes from a large variety of environments, such as water, air and sediments, whenever qPCR is needed for gene quantification.

## Data availability statement

The datasets presented in this study can be found in online repositories. The names of the repository/repositories and accession number(s) can be found in the article/Supplementary material.

## Author contributions

XH: Conceptualization, Formal analysis, Investigation, Methodology, Writing – original draft, Writing – review & editing. KB: Writing – review & editing. HB: Writing – review & editing. BF: Writing – review & editing. BS: Writing – review & editing, Methodology. AF: Writing – review & editing, Funding acquisition, Supervision, Writing – original draft.
