# Supplementary material for: Synthetic oligonucleotides as quantitative PCR standards for quantifying microbial genes
Source: Front Microbiol. 2023 Oct 24;14:1279041. doi: 10.3389/fmicb.2023.1279041 (PMC10627841; doi:10.3389/fmicb.2023.1279041)
Supplement: Supplementary file 1 [file Data_Sheet_1.pdf]

Synthetic oligonucleotides as quantitative PCR standards for quantifying microbial genes

Xingguo Han[1,*], Karin Beck[2,], Helmut Bürgmann[2], Beat Frey[1], Beat Stierli[1], Aline Frossard[1,*]

1. Forest Soils and Biogeochemistry, Swiss Federal Institute for Forest, Snow and Landscape Research (WSL), Birmensdorf, Switzerland

2. Eawag, Swiss Federal Institute of Aquatic Science and Technology, Kastanienbaum, Switzerland

* For correspondence: xingguo.han@wsl.ch; aline.frossard@wsl.ch

Forest Soils and Biogeochemistry, Swiss Federal Institute for Forest, Snow and Landscape Research (WSL), Zürcherstrasse 111, 8903 Birmensdorf, Switzerland

Tel: +41 44 739 28 07

Supplementary Tables

Supplementary Table S1. Sequences of synthetic DNA fragments used as qPCR standards. The red sequences are corresponding to the primer sequences containing only known nucleotides of A, T, C, and G. The top six genes (bold fonts) were compared with both synthetic DNA standard and plasmid DNA standard.

**Targetted phylogenetic group / Processes invovled**	**Gene**	**Sequence (5'-3')**	**Most similar organism (Blast result from NCBI)/ Percentage Identity**
**Bacteria universal**	**16S rRNA gene**	ACGAGTCCAGACTCCTACGGGAGGCAGCAGTGGGGAATCTTGCACAATGGGCGAAAGCC<br>TGATGCAGCGACGCCGCGTGAATGATGAAGGCCTTTTCGGGTTGTAAAGTCCTTTCATTTG<br>GGAAGAAGGGAGTAAAGACTAATAATTTTGCTCATTGACGGTACCCGAAGAAGAAGCAC<br>CGGCTAACTCCGGTGCCAGCAGCCGCGGTAATACGGAGGGTGCGAGCGTTGTTCGGAATT<br>ACTGGGCGTAAAGCGCGCGTAGGCGGTTTGGTAAGTCAGATGTGAAATCCCGGGGCTCA<br>ACCGTCGGAACTGCATTTGAAACTGCCAAGCTTGAGTGCGGGAGAGGGGAGTGGAATTC<br>CAGGTGTAGCGGTGAAATGCTGTAGATATCGGGAGGAACACCGGTGGCGAAGGCGGCTC<br>CCTGGACCGTTACTGACGCTGAGGCGCGAAAGCGTGGGGAGCAAACAGGATTAGATACC<br>CTGGTAGTCCACGCTGTAAACGA	*E. coli* (Gammaproteobacteria)/90.56%<br><br>*Vibrio sp.* (Gammaproteobacteria)/90.24%
**Fungal universal**	**ITS**	AACGGATCTCTTGGTTCTCCCATCGATGAAGAACGCAGCGAATTGCGATAAGTAATGTGA<br>ATTGCAGAATTCAGTGAATCATCGAATCTTTGAACGCACCTTGCGCCCTTGGGTATTCTCA<br>AGGGCATGCCTGTTTGAGTGTCATAACAATCTCGACTCTCAGCTTTTTTGAAAGAGAAGG<br>CTGCTTGGAGTCGGTGATGGGCGTTTGCCATACTGTCACTCGTCTGGCTCGCCTGAAATGC<br>ATCAGTGGCTCCTTCTGTGGAGACCGGTCTGACTATGTGTGATAATTTGATCGCATAGGAT<br>GTGTGTCCCTTGCGGAGACACAACCGGACTTGCTGTTAGGAGGTTGCTTCTAACCCGGAA<br>TGCTTTGTTGCGCCCATGGCATCTTACTTGTCTGACCTCAAATCAGGTAGGACTACCCGCT<br>GAACTTAAGCATATCAATAAGCGGAGGAGAAAAAACTAACAAGGATTC	*Tilletia vittata* (Basidiomycota)/94.94%

**Methane production**	***mcr*A**	TCATACATGTCAGGTGGTGTAGGATTCACACAATATGCTACAGCAGCATACACCGACGAC<br>ATCCTCGACGACTTCACATACTACGGTAATGACTACGTAGAGGACAAATACGGTGGACTC<br>GGAACTAAGGCAACAAACACCATGGACGTAGTACAAGACATTGCAACCGAAGTTACACT<br>CTACGGACTTGAACAGTACGAGGAATACCCAGCATTATTAGAAGACCACTTTGGTGGATC<br>ACAGAGAGCAGCAGTTGCTGCAGCTGCAGCTGGTTTTTCAACATCTATTGCAACAGGTAA<br>TGCTCAAACGCTGGTCTCAACGGTTGGTACTTATCACAAATACCTACACAAAGAATACCA<br>CGGCAGACTTGGATTCTACGGTTACGACTTGCAGGACCAATGTGGTGCTTCAAACTCCTTC<br>TCAATAAGAAGCGACGAAGGTTTACCACTTGAACTACGTGGACCTAACTACCCTAACTAC<br>GCAATGAACGTTGGTCACCA	*Methanococcus* (Euryarchaeota)/85.40%
**Methane oxidation**	***pmo*A**	TTCACTTCATGCTGACCGTGGGCGACTGGGACTTCTGGGCCGACTGGAAAGATCGTCGTT<br>TGTGGCCGACGGTCACGCCGATCGTCGGCATCACCTTCCCGGCGGCGGTGCAAGCGGTGC<br>TGTGGGATCGTTTCCGTCTGCCTTTGGGCGCCACCTTCTGCGTTCTGGGCTTGTTGATCGG<br>CGAATGGGTCAACCGCTACTTCAACTTCTGGGGCTGGACCTACTTCCCGATCAACTTCGTG<br>TTCCCGGCTACGATGATGCACAGGCGCGATCGTTCTGGACGTGGTGCTGATGCTGTCCGG<br>CAGCTTCATGATCACCGCCATTGTTGGCGGGTTGGCCTGGGGTTTGTTGTTCTACCCGAGC<br>AACTGGCCGATCATCGCCCCGTTCCACGTGCCGGTCGAATACAACGGTATGCTGATGACG<br>GTGGCCGATTTGCTCGGTTACCACTACGTCCGTACCGGTATGCCGGAATACATCCGCATG<br>GTCGAGCGCGGCACCCTGCGCACCTTCGGTAAGGACGTGGCGCCGGTGTCGGCGTTCTTC<br>TCGGCCTTCATGTCCATGCTGATGTACTT	*Methylomarinovum caldicuralii* (Gammaproteobacteria)/83.19%
**Nitrogen fixation**	***nif*H**	GGTCAGAAGATCATGATCGTCGGCTGCGACCCGAAGGCTGACTCCACCCGCCTGATCCTG<br>CACGCCAAGGCGCAGAACACCGTGATGCACCTGGCCGCCGAAGCCGGCTCCGTGGAAGA<br>TCTGGAGCTCGAGGACGTGCTGAAGGTCGGCTACGGCGGCATCAAGTGCGTCGAGTCCGG<br>TGGTCCGGAGCCGGGCGTCGGCTGCGCCGGCCGTGGCGTTATCACCGCCATCAACTTCCT<br>GGAAGAGAACGGCGCCTACGACGACGATCTCGACTTCGTCTCCTACGACGTGCTGGGCGA<br>CGTGGTCTGCGGCGGCTTCGCCATGCCGATCCGCGAAAACAAGGCTCAGGAAATCTACAT<br>CGTCATCTCCGGCGAGATGATGGCGATGTACGCCGCCAACAACATCTCCAAGGGCAT	*Azospirillum sp.* (Alphaproteobacteria)/91.55%
**N_2O reduction**	***nos*Z**	ACCTTCGACGGCCGCGGCAACGCCTACACCACGCTGTTCATCGACAGCCAGGTGGTGCAA<br>GTGGAACATCGACGACGCCATGCGCGCCTACAAGGGCGAGAAGGTCAACTACATCAACC<br>AGAAGCTCGACGTGCACTACCAGCCGGGCCACAACCACGCCTCCCTGAGCGAGACCAGC<br>GAAGCCGACGGCAAATGGCTGGTGGTGCTGTGCAAGTTCTCCAAGGACCGCTTCCTGCCC<br>ACCGGCCCGCTGCACCCGGAGAACGACCAGTTGATCGACATTTCCGGCGACGAGATGAA<br>GCTGGTGCACGA	*Pseudomonas sp.* (Gammaproteobacteria)/93.85%
Ammonia oxidation	archaeal *amo*A	TGTTTCTACAATATGGTAATGGTCTGGCTTAGACGATGTACTCACTACTTATTCATAGTAG<br>TAGTTGCAGTTAACTCAACACTGTTAACAATTAATGCAGGAGACTACATTTTCTATACTGA<br>CTGGGCTTGGACTTCGTTCACGGTATTTTCAATATCGCAAACGTTGATGCTTTGCGTAGGT<br>GCAACATATTACCTGACATTTACAGGTGTTCCAGGAACAGCAACGTATTACGCCCTAATT<br>ATGACAGTATACACATGGGTAGCAAAAGGCGCATGGTTTGCACTTGGTTATCCATATGAC	Uncultured archaeon/96%

		TTCATTGTAACACCAGTTTGGTTACCATCAGCAATGTTGCTTGATTTGGCCTATTGGGCGACAAAGAAGAACAAGCACTCCTTGATACTGTTTGGCGGGGTACTGGTAGGAATGTCTTTACCATTATTCAACATGGTAAACCTGATAACAGTAGCAGACCCACTAGAAACGGCATTCAAATACCCAAGACCAACATTGCCACCATACATGACACCAATAGAACCTCAAGTAGGTAAATTCTATAACAGTCCAGTAGCACTGGGTGCAGGTGCGGGTGCAGTTTTGTCAGTTACATTTACAGCGTTAGGTTGTAAACTAAACACTTGGACATACAGATGGATGGCCGCAAGGGCGAATTCGTT	
Ammonia oxidation	bacterial *amo*A	TGAGTGGCTGACACGTTACTGGGGATTCTACTGGTGGTCACACTACCCCATCAACTTCGTAATACCGGGCATTATGATTCCGGGTGCGCTGATGCTGGACATCACGCTGTATCTGACACGCAACTGGATGGTCACGGCTCTGGTTGGAGGTGGAGCCTTCGGTCTGCTGTTCTATCCGGGTAACTGGCCGATTTTTGGACCAACCCACTTGCCAATCGTTGTAGAAGGCATATTGCTGTCGATGGCTGATTACATGGGATACCTGTATGTTCGTACAGGTACACCCGAGTATGTTCGTCTTATTGAACAAGGTTCACTGCGTACCTTTGGTGGTCACACCACAGTTATTGCAGCATTCTTCTCTGCGTTCGTATCAATGTTGATGTTCACCGTCTGGTGGTACCTTGGAAAAGTTTACTGCACCGCCTTCTTCTACGTTAAAGGTCCAAGAGGTCGGATCACACAGAACAATGATGTTACCGCATTCGGCGAAGAAGGCTTTCCAGAGGGGATCAAATAAAATGGGTATCA	*Nitrosomonas europaea* (Betaproteobacteria) /94.73%
N_2O production	*nor*B	TTCTTGCTCGGCCACCAGGGCTATGAGTATGTCGACCTGGGCCGGCTGTGGCAGATCGGCAAGTTCGCCGGCATCCTGATCTGGCTGGTGCTGATGATGCGCGGCATCCTGCCGGCGCTGCGCGCGCGCGGCACCGACCGCAACCTGCTGGCGCTGCTGACCTCGTCGGTGGTGGCAATCGGCCTGTTCTACGGCGCGGGCCTGGCCTACGGCGAACGCACCAGCCTGACGGTGATGGAGTACTGGCGCTGGTGGGTGGTGCACCTGTGGGTTGAAGGCTTCTT	*Cupriavidus necator* (Betaproteobacteria) /98.59%
Organic phosphorus hydrolysis	*pho*D	CAG**TGGGATGATCACGAGGT**GACCAACAACTGGTGGCCGGGCGAGCCGCTGACCCGCGCCGAGCATGCGCGCAAGAACTACGTCGATCGCAACGCACTGCTGCTGGCGGCGCGGGCGAGCCGCGCGTTCCACGAATATATGCCGATGCGCTTCACCCAGGCCGAGCCGGGGCGCGTCTACCGCAAGATCTCCTACGGACCGCTGCTCGACATTTTCATGCTCGACATGCGCAGCTATCGCGGACCCAACGGCGAAGGCCTGGAGGAAAGCTATGGGCCGGCGGCGTACTTCCTCGGCCTGACCCAGGTGGCTTGGCTCAAGCGCGAGCTGATGACGTCGCAGGCGACC**TGGAAGGTGATCGCCCAG**GACATGCCGAT	Uncultured bacterium/98.67%
Nitrite reduction	*nirS*	ACGAGCACCCGGAGTTCAT**CGTCAACGTGAAGGAAACCGG**CAAGGTCATGCTGGTCAACTACAAGGACATCGACAACCTCACCATCACCAGCATCGATGCCGCGCCGTTCCTGCATGACGGCGGCTGGGACAGCAGCCACCGCTACTTCATGACGGCCGCCAACAACTCCAACAAGGTTGCGGTGATCGACTCCAAGGACCGCAAGCTGGCGGCCCTGGTCGATGTCGGCAAGATCCCGCACCCGGGCCGTGGCGCCAACTTCGTGCATCCGAAGTACGGCCCGGTGTGGGCCACCAGCCACCTGGGCGACGACAGCATCTCGCTGATCGGCACGCGATCCGAAGAATCCAAGTCCGCCAAGTACAAGGAGCACACCTGGAAGGAGGTCGCCACGCTGCAGGGCCAGGGCGGCGGCTCGCTGTTCA**TCAAGACCCATCCGAAGTC**CAAGCACCTGTACGTCGACA	*Pseudomonas aeruginosa* (Gammaproteobacteria)/90.32%

Nitrite reduction	*nirK*	TCTCGGGCATGAACGGCGCG**ATCATGGTGCTGCCGCG**CGACGGTCTGAAGGACGACAAGGGCAAGGCGCTGACCTACGACAAGGTCTACTACGTCGGCGAGCAGGACTTCTACGTCCCGCGGGACGAGAACGGCAAGTTCAAGAACTACGAGACCCCGGGCGAAGCCTATGACGACACCGTCGAGGTGATGCGCACGCTGACCCCGACCCACGTCGTCTTCAACGGCGCGGTCGGCGCGCTGACCGGCGACAATGCGCTGACGGCCAAGGTGGGCGAGAACGTGCTGATCGTCCACTCGCAGGCCAACCGCGACACCCGCCCGCACCTGATCGGCGGCCATGGCGACTATGTCTGGGAGACCGGCAAGTTCGACAACCCGCCCGAGCTCGACCTGGAAACCTGGTTCATTCGCGGCGGCTCGGCCGGGGCTGCGCTCTACACGTTCCAGCAGCCCGGCGTCTACGCCTATGTG**AACCACAACCTGATCGAGGC**CTTCGAACTCGGCGCGGCCG	*Chelatococcus sp.* (Alphaproteobacteria)/88.85%
Nitrite oxidation	*nxrB*	TCTTCTCGAAGGGGCAGGAG**TACATGTGGTGGAACA**ACGTGGAAACCAAGCCGTACGGCGGGTATCCCCAGTTCTACGACGTGAAGATCACGCAGCTCATCGAGCAGGTGAACCCGGGGGGCCAGGTGTGGAACGTCCGGGTGGGACGCAAGCACCATGCGCCGTACGGGGTGTTCGAAGGGATGACCATTTTCGACGCCGGGGCCAAGGTCGGCCAGGCGGCGATCGGGTACATCCCGACGGACCAGGAATGGCGGTTCGTGAACATCTATGAGGACACGGCGACCTCGATGCGGGCCCTGGTGGAGGGCATCGACAAGTCGGGCTTCACGCGCGACGAACCGTGGCGGCTGTCCGGCAGCAGCTTGCCGGAGCATGAGACGTTCTTCTTCTATCTGCAGCGGATCTGCAACCACTGCACCTATCCGGGCTGCCTGGCAGCCTGCCCGCGCAAGGCGATCTACAAGCGGCCGGAAGACGGCATCGTGT**TGATCGACCAGAACCG**GTGCCGCGGGTACAAGAAGT	*Nitrospira defluvii* (Nitrospirota)/96.38%

Supplementary Table S2. Sampling sites and soil pH, total carbon (TC), total organic carbon (TOC), total nitrogen (TN), and DNA. m.a.s.l.: meters above sea level. Mean and standard deviation values are from biological replicates (n=3).

Sample name	Sites	Coordinates	Altitude (m.a.s.l.)	DNA concentration (ng/µl)	Absorbance ratios from Nanodrop		TC (%)	TOC (%)	TN (%)	pH
					A260/A280	A260/A230				
A	Vordemwald	N47°16.402', E007°53.204'	530	407.94±30.23	1.91±0.03	1.46±0.44	14.13±3.12	14.13±3.12	0.81±0.15	2.83±0.09
B	Alpthal	N47°02.874', E008°42.757'	1159	391.44±74.09	1.95±0.01	1.86±0.17	13.75±3.47	13.75±3.47	0.76±0.15	4.06±2.83

C	Celerina	N46°29.523', E009°53.424'	1844	239.80±39.57	1.94±0.01	1.89±0.13	5.16±2.39	5.16±2.39	0.22±0.09	3.61±0.47
D	Umbrail Meadow	N46°33.219', E010°26.057'	2336	409.33±223.97	1.92±0.02	1.85±0.11	17.31±1.17	10.59±2.37	1.06±0.26	6.94±0.26
E	Umbrail Ruins	N46°32.932', E010°25.450'	2715	132.45±103.03	1.96±0.01	0.77±0.18	3.79±2.35	2.58±1.42	0.24±0.13	6.64±0.70

Supplementary Table S3. Cq values of the least diluted standard of each target gene. n=3.

Gene	Synthetic DNA standard		Plasmid DNA standard	
	**Cq**	**Highest standard (copies per µL)**	**Cq**	**Highest standard (copies per µL)**
16S rRNA gene	9.74 ± 0.01	1.83E8	9.49 ± 0.37	2.50E8
ITS	12.59 ± 0.17	1.93E7	10.89 ± 0.00	1.83E7
*mcr*A	4.92 ± 0.02	1.83E8	7.79 ± 0.08	2.85E8
*pmo*A	15.74 ± 0.14	1.60E6	17.49 ± 0.14	1.70E6
*nif*H	10.03 ± 0.05	2.19E7	12.90 ± 0.43	1.84E7
*nos*Z	6.39 ± 0.20	2.95E8	10.81 ± 0.07	1.18E8

Supplementary Figures

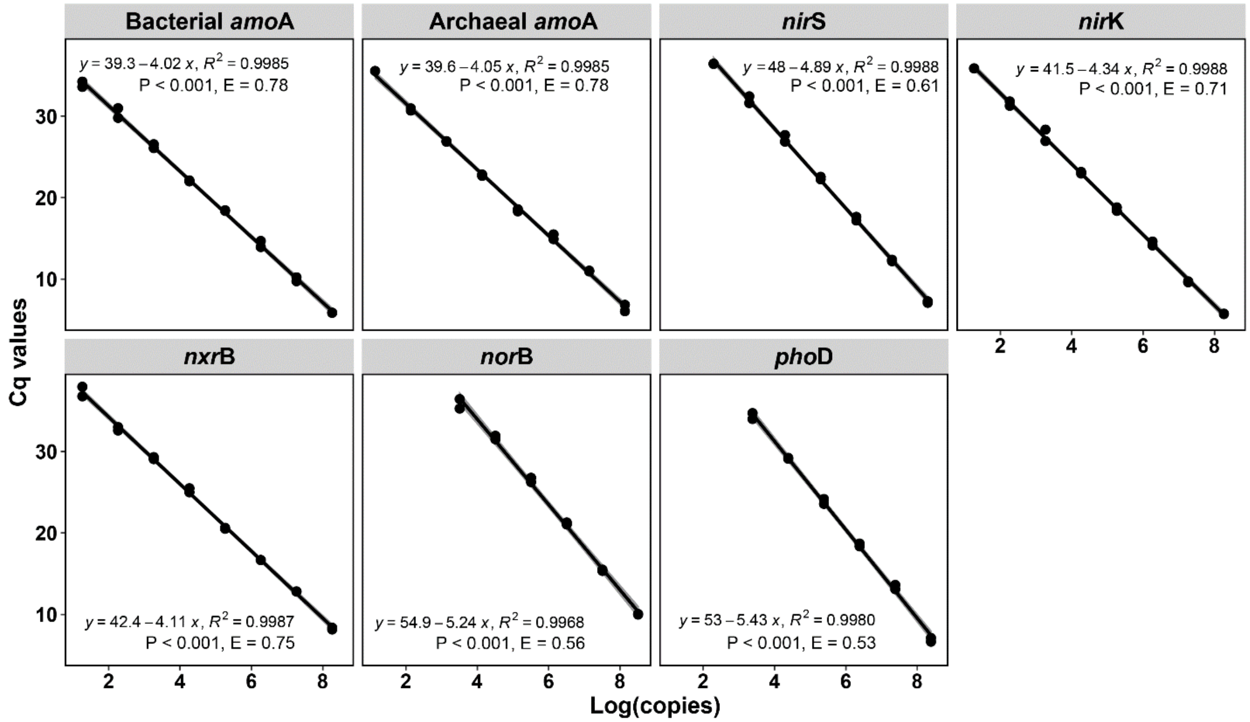

Supplementary Fig. S1. qPCR standard curves using synthetic DNA as standards. Standards were diluted by 10 times for each step from 10^8 to 10^1 copies per µl. R^2 is the coefficient of determination. P < 0.001 indicates the significance of the linear regression. Gene copy numbers (copies per dry gram soil) were log10-transformed. E = (10−1/slope) – 1. n = 3.

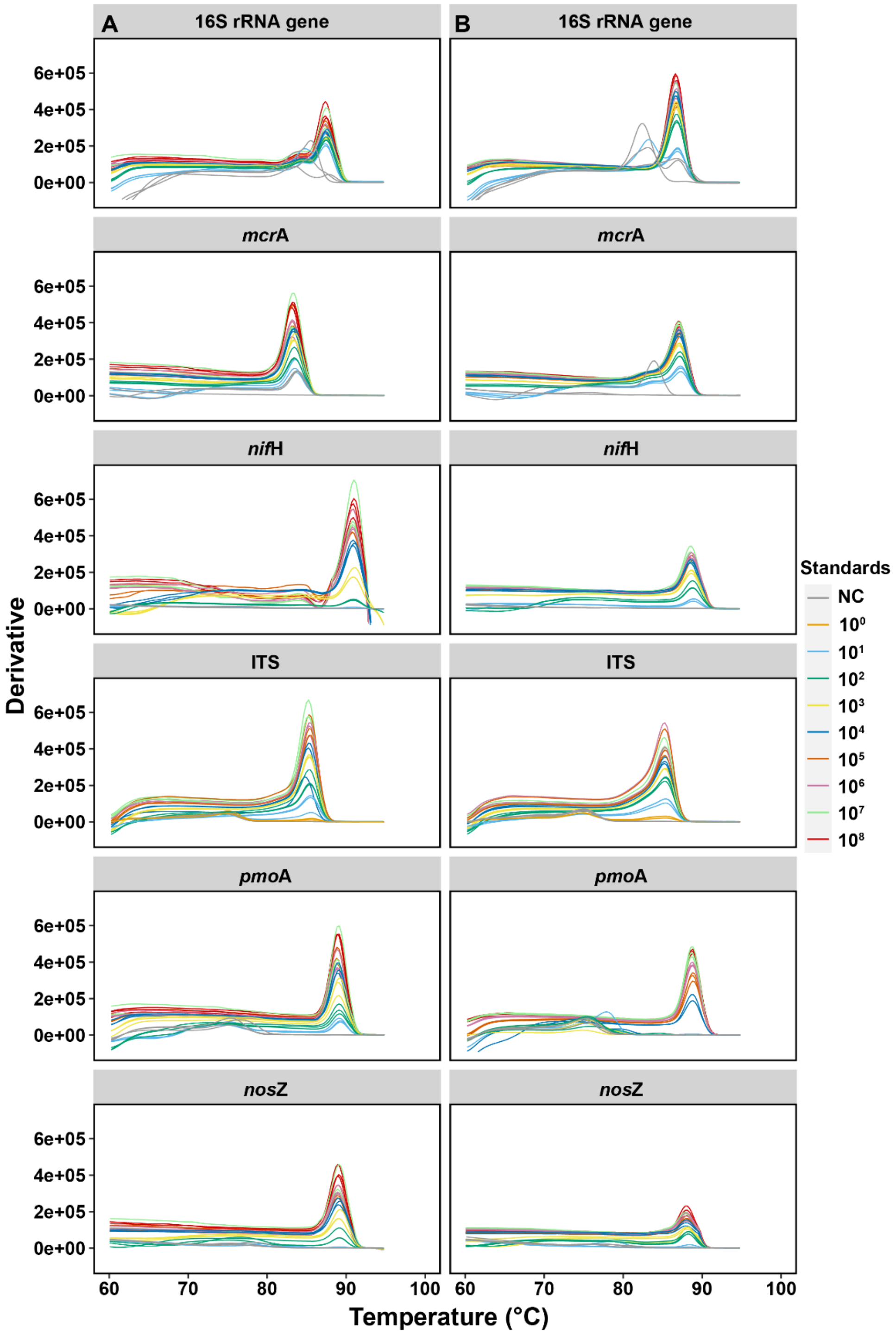

Supplementary Fig. S2. **Melting curves of each target gene by serial dilutions between synthetic DNA (A) and plasmid DNA (B) standards.** Each qPCR reaction (10 µl total) contained 2 µl of DNA template (standard). Standards were diluted by 10 times for each step from 10^8 to 10^1 copies per µl. NC: negative control.

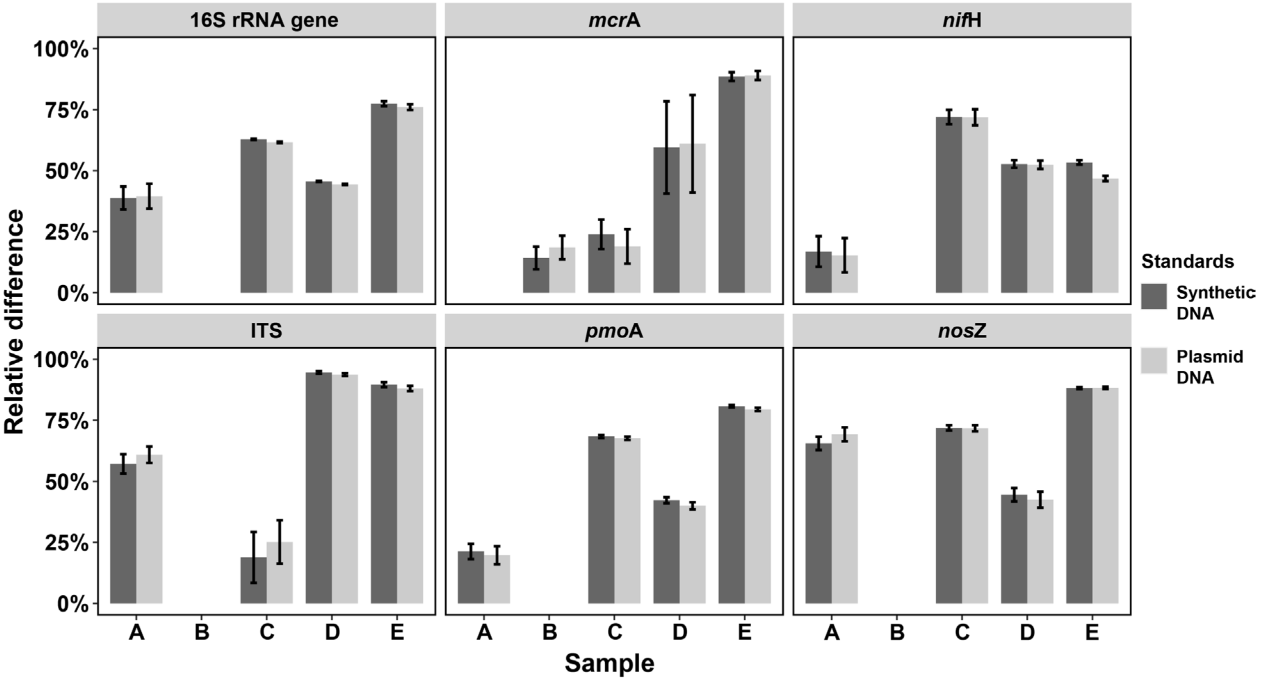

Supplementary Fig. S3. Comparisons of variations of gene copy numbers among soils normalized to copy numbers of the soil with the highest copy numbers for each gene, calculated for both synthetic and plasmid standards. All genes had the highest copy numbers per dry gram soil in soil B from both synthetic and plasmid standards, except for *mcr*A in soil A. Significant differences in gene copies between plasmid DNA and synthetic DNA standards were done based on T-test test by the function compare_means() from the R package ggpubr. P-Values were adjusted by the "bonferroni" method. There were no significant differences in relative differences between the two standards per soil sample across all the tested genes.
